# Editorial: Effects of perinatal opioid exposure—volume II

**DOI:** 10.3389/fped.2024.1396595

**Published:** 2024-04-05

**Authors:** Loretta P. Finnegan, Henrietta S. Bada

**Affiliations:** ^1^ Finnegan Consulting, LLC, Avalon, NJ, United States; ^2^ Department of Pediatrics, College of Medicine, University of Kentucky, Lexington, KY, United States

**Keywords:** perinatal opioid exposure, neonatal abstinence syndrome, methadone, clonidine, long-term outcome, toxicology, ECMO, imaging

**Editorial on the Research Topic** Effects of perinatal opioid exposure—volume II

## 1 Introduction

This second volume of the research topic on the Effects of Perinatal Opioid Exposure covers issues that address the various aspects of managing individuals during pregnancy, considering not only the treatment of opioid use disorder (OUD) but also the many factors that may affect the outcomes of pregnancy and infants with in-utero drug exposure (IUDE). Topics in this volume include preclinical studies, management of substance use disorder (SUD) during pregnancy, management of infants with prenatal exposure, long-term outcome studies that take into consideration factors such as parenting and psychological distress, examination of executive functioning and the trajectory of behavioral problems, and support for a recommendation to evaluate for individual and various other factors that may influence outcomes.

## 2 Preclinical studies

Clinically, oral opioid therapies for prenatal opioid exposure (POE) with the development of neonatal abstinence syndrome (NAS) are a standard of care, with morphine being the most commonly used medication. A non-opioid agent, clonidine, has recently been used for treatment of infants with NAS. However, data regarding the cellular and molecular effects of these treatments on the developing brain are still lacking. To address this gap in knowledge, Sithisarn et al., determined the effects of morphine or clonidine on the cell death of neonatal cortical explant cultures from Sprague Dawley rats after in-utero exposure to oxycodone. Explants from the prefrontal cortex (PFC) demonstrated greater cell death after prenatal treatment with oxycodone and postnatal treatment with morphine compared to treatment with clonidine. The PFC is vital for controlling higher-order executive functions such as behavioral flexibility, learning, and working memory.

Chin et al. defined the effects of POE on whole-brain functional connectivity and white matter injury using quantitative whole-brain structural and functional MRI in an established rat model of POE. Decreased connectivity in cortical-cortical and cortico-basal ganglia circuitry was particularly prominent with large effect sizes. These data support that POE reduces brain-wide functional connectivity as well as the microstructural integrity of major white matter tracts. Altered neural circuitry, dysregulated network refinement, and diffuse network dysfunction have been implicated in the executive function deficits that are common in children with POE. Functional brain connectivity may serve as a translatable biomarker in children with POE.

## 3 Clinical investigations

Many healthcare providers lack training in screening for or treating SUD during pregnancy. The proliferation of punitive policies toward SUD has led to decreased prenatal care, no improvement in birth outcomes, and a disproportionate impact on Black, Indigenous, and other families of color. Barber and Terplan described the principles of care during pregnancy from an obstetrician-gynecologist perspective related to SUD, including the need to understand the unique barriers of pregnancy-capable persons, care for the dyad, person-centered language, and the high risk of mortality in the postpartum period with drug overdose being one of the leading causes of maternal death in the United States.

The use, misuse, and abuse of substances, particularly opioids, is an ongoing public health concern in this country and around the world. Resources to assist perinatal health professionals with this very complex subject are limited. Jones provides up-to-date information on the selection of monitoring protocols, the specifics of appropriate testing methodologies, and the interpretation of toxicological findings. A better understanding of these concepts will enable perinatal healthcare professionals to be a voice for the voiceless in order to protect and enrich lives during this unprecedented opioid epidemic.

Since the first use of methadone to treat OUD in pregnancy in the 1970s, there has been a long, controversial, and confusing history of studies, regulatory actions, and changes in practice that have clouded an accurate perception of methadone's use in pregnancy. McCarthy and Finnegan trace this history with a focus on the effect of methadone exposure during pregnancy on NAS. A new laboratory measure, the serum methadone/metabolite ratio, has provided a tool for documenting the profoundly dynamic nature of perinatal metabolism. The continuous induction of metabolic enzymes during pregnancy requires dose adjustments and changes in dosing frequency. The concept of “fetal methadone dosing” emphasizes that the relative stability of methadone levels in the fetus is an important consideration for methadone dosing in pregnancy.

The sharp increase in NAS cases has resulted in increased healthcare expenditures, resource utilization, and hospitalization of infants requiring pharmacotherapy. To mitigate the consequences of maternal-infant separation during pharmacological treatment, the Eat, Sleep, and Console (ESC) tool has become popular and is promoted as a novel method that focuses on the maternal/infant dyad with the resultant reduction of treatment duration and hospital stay. Gomez Pomar reviewed the studies on ESC and highlighted the differences among the studies. The majority were based on quality care initiatives with conflicting results. Although staff training has been proposed and the interventions of ESC have been defined, there still exists a lack of standardization of this practice, specifically with regard to the type of associated non-pharmacological practices as well as the reports of its short- and long-term outcomes, which may be attributable to a lack of randomized research trials. In a recent large multicenter trial using cluster randomization, infant follow-up was limited to 3 months post-discharge with no standard infant assessment.

The incidence of in-utero drug exposure (IUDE) and the use of neonatal extracorporeal membrane oxygenation (ECMO) have both increased over the past decade. There are no studies of infants with IUDE who required a life-saving procedure such as ECMO. Walther et al. reported that infants with IUDE had greater use of sedative and analgesic adjuvant medications during ECMO than infants with no IUDE on ECMO. Trend results indicated that post-ECMO feeding complications and total hospital stay were also greater in the IUDE-ECMO group. These findings illustrate the complex influence of prenatal drug exposure on neonatal patient care and warrant the development of clinical care strategies optimized for this unique patient group.

During the current opioid epidemic, opioids are commonly used with other substances such as tobacco and, more recently, the increase in methamphetamine has been selective to opioid use, particularly in rural regions. Wouldes and Lester provide a comprehensive review of the perinatal effects of the use of opioids and/or methamphetamines during pregnancy highlighting these effects on pregnant individuals and their infants. The characteristics of the women in both the opioid and methamphetamine studies were associated with poor maternal health, higher rates of mental illness, trauma, and poverty. Cardiovascular disease is not uncommon among women with substance use disorders, including opioid, methamphetamine, cocaine, alcohol, cannabis, or polydrug use. Women who used opioids and methamphetamines were reported to have poor maternal health, and rates of mental illness, trauma, and poverty. Infant outcomes that differed between opioid and methamphetamine exposure included variations in neurobehavior at birth which could complicate the diagnosis and treatment of neonatal opioid withdrawal. Given the complexity of OUD in pregnant individuals and the increasing co-use of these opioids with methamphetamine, future studies need to address the many confounders of perinatal outcomes and employ neurodevelopmental markers at birth that may help predict long-term neurodevelopmental outcomes.

## 4 Long-term outcome studies

The review by Yen and Davis elucidates the many reasons why very little is known about the immediate and long-term outcomes of these children with NAS which include: (1) barriers to maintaining short-term and long-term follow-up; (2) unclear mechanisms by which prenatal opioids affect the developing brain; (3) the multiplicity of psychosocial factors that affect child development, and the varying degrees of deficits in different domains that are reported following prenatal opioid exposure; and (4) the non-uniformity of standardized tests administered at follow-up. Although not all of these factors are addressed or controlled for in all follow-up studies, the information would make clinicians and researchers aware of the possibility of lasting effects of perinatal opioid exposure.

Sarfi et al. prospectively followed mothers with OUD who were receiving opioid maintenance therapy (OMT), their children, and a comparison group of mothers with no history of substance use and their children. From the trajectories of maternal parenting distress and mental health, mothers on OMT had higher parenting distress and psychological distress than the comparison group. Parenting distress did not seem to affect the subscale of dysfunctional parent-child interactions or the subscale difficult child. Few mothers needed clinical intervention for psychological distress. Children of mothers on OMT had significantly higher levels of behavioral problems noted at 4.5 years of age than did comparison children, and these problems persisted to 8 years of age. However, problem scores decreased by 8 years in the comparison children. The long-term direct effects of prenatal opioid exposure on behavior problems appear to be modest in what appears to be a stable caregiving environment while receiving OMT.

Spowart et al. evaluated children with methadone exposure at ages 8–10 years, and a control group matched for gestational age, birth weight, and socio-economic status. Results from the administration of a battery of tests indicated no differences between exposed and non-exposed children as to the proportion of emotional, conduct, peer relationships, total difficulties or prosocial problems. However, a marginally higher proportion of exposed children had hyperactivity problems. In terms of executive regulation, the exposed children were significantly worse on indices of behavioral, emotional and cognitive regulation, and on the global executive composite. However, the effect of methadone was reduced with higher tobacco use. The study highlighted the importance of controlling for confounders in the determination of the effects of prenatal methadone exposure. These findings in school-aged children indicate a modest effect of methadone exposure on executive regulation.

